# In This Issue: Volume 116, Issue 5, May 2025

**DOI:** 10.1111/cas.70084

**Published:** 2025-05-01

## STAT3 Inhibition Prevents Adaptive Resistance and Augments NK Cell Cytotoxicity to KRAS^G12C^ Inhibitors in Nonsmall Cell Lung Cancer

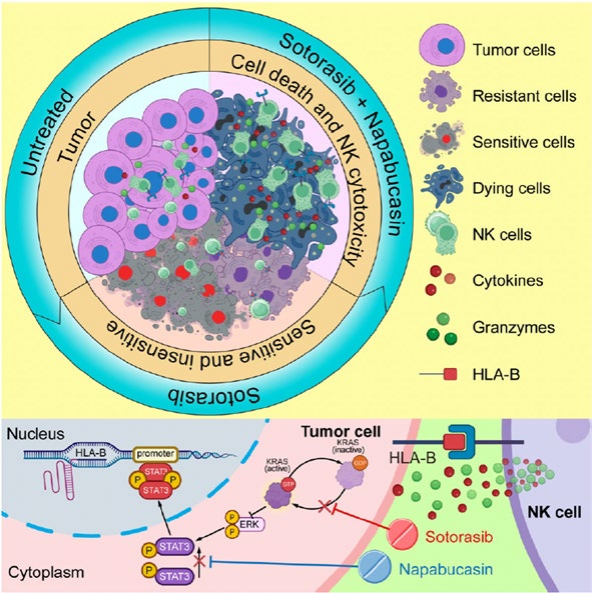

KRAS^G12C^‐mutant non‐small cell lung cancer (NSCLC) presents a significant challenge in cancer treatment due to the rapid onset of resistance to targeted therapies. Although KRAS^G12C^ inhibitors, like sotorasib, show initial clinical success, patients often experience adaptive resistance, meaning the cancer becomes less responsive to treatment, limiting long‐term effectiveness.

To address this challenge, researchers explored combining sotorasib with napabucasin, a drug that inhibits STAT3, a protein involved in cancer survival and immune suppression. Through high‐throughput screening of 423 compounds, the researchers identified that napabucasin worked synergistically with sotorasib to suppress tumor growth in both sensitive and drug‐resistant KRAS^G12C^‐mutant NSCLC cell lines. Functional assays demonstrated that the combination not only enhanced tumor growth inhibition but also promoted the activation and infiltration of natural killer (NK) cells within the tumor. NK cells play a crucial role in the body's immune defense against cancer, and their activation is critical for effective tumor clearance.

At a molecular level, the study revealed that blocking KRAS^G12C^ unexpectedly activated STAT3, which helps tumors resist treatment by weakening the immune system. Napabucasin counteracted this effect by preventing STAT3 from binding to HLA‐B, a gene that produces signals inhibiting NK cell activity. By reducing these signals, napabucasin enhanced NK cell function, making the immune system more effective at attacking tumors.

This research sheds light on an important mechanism of adaptive resistance to KRAS^G12C^ inhibitors, demonstrating that STAT3 activation supports tumor regrowth under KRAS inhibition. By blocking STAT3 and the associated suppression of NK cell activity, the study offers a new combination therapy approach that not only targets the cancer cells more effectively but also boosts the immune system's ability to fight the tumor. These findings provide strong evidence that combining KRAS^G12C^ and STAT3 inhibitors could represent a promising strategy to improve treatment responses and overcome resistance in KRAS^G12C^‐mutant NSCLC.

https://onlinelibrary.wiley.com/doi/10.1111/cas.70017

## Single‐Nucleus RNA Sequencing and Spatial Transcriptomics for Squamous Cell Carcinoma Arising From Ovarian Mature Teratoma

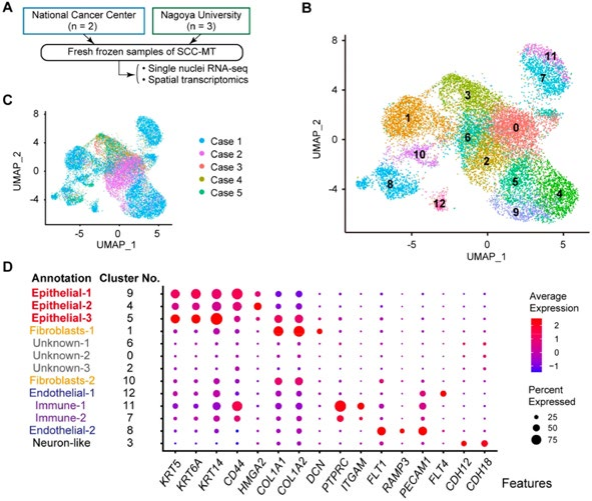

Cancer is a disease that causes uncontrolled growth of cells. One of the leading forms of cancer that affects women is ovarian cancer. Squamous cell carcinoma arising from mature teratoma (SCC‐MT) is a rare and aggressive type of ovarian cancer. It develops from a usually benign type of ovarian tumor called a mature teratoma. Because SCC‐MT is so rare, and doctors don't fully understand what makes it grow, we don't yet have a standard treatment.

In this study, researchers used biological techniques such as single‐nucleus RNA sequencing and spatial transcriptomics to map which genes are active in individual cells and where those cells are located within the tumor. The researchers identified three groups of cells being linked with SCC‐MT. Among them, one group of cancer cells is closely associated with skin cells, supporting the idea that SCC‐MT arises from teratomas. These skin‐associated cancer cells activate powerful growth‐related pathways, which may help explain why this rare ovarian cancer can be so aggressive.

The researchers also found that a gene called Krüppel‐like factor 5 (*KLF5)*, which normally helps regulate the growth of skin cells, was highly active in the cancer cells in SCC‐MT. When they reduced the activity of *KLF5* in lab‐grown cancer cells, the cells grew more slowly, and many died. They also studied a microRNA called miR‐145‐5p, which normally keeps KLF5 levels in check. In cancer, this small RNA molecule was less active, which may help explain why KLF5 levels were high.

Because this type of cancer is so rare, more research is still needed. Utilizing complex biological techniques can help reveal various mechanisms linked with SCC‐MT. Understanding the connections between skin cell behavior, gene regulation, and cancer growth not only gives us a clearer picture of how SCC‐MT develops, but also points to *KLF5* as a promising target for future treatments.

https://onlinelibrary.wiley.com/doi/10.1111/cas.70022

## SPOP Suppresses Hepatocellular Carcinoma Growth and Metastasis by Ubiquitination and Proteasomal Degradation of TRAF6

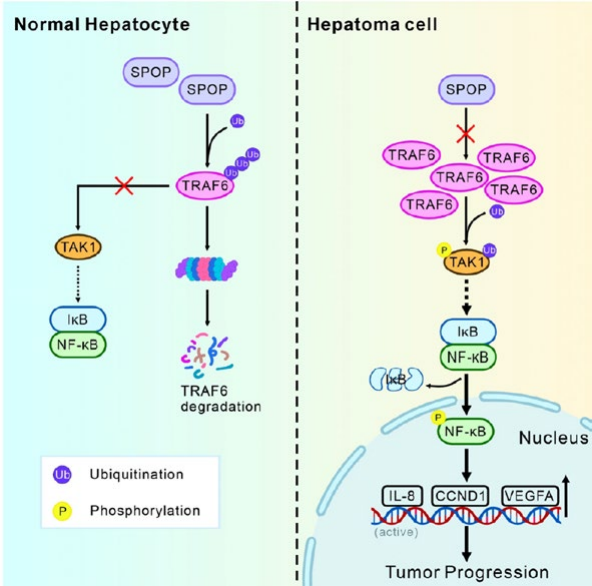

Liver cancer or hepatocellular carcinoma (HCC) is one of the most common and deadly cancers. Many factors, including hepatitis infections, alcohol use, and metabolic diseases, can contribute to its development. The treatment options for advanced HCC remain limited, making it crucial to understand the underlying mechanisms driving the disease.

TRAF6 is a protein that activates the NF‐κB cell signaling pathway, which can promote cancer cell growth and spread. This study found that TRAF6 levels are significantly higher in HCC tissues compared to healthy liver tissue. Under normal conditions, SPOP, a protein involved in cellular regulation, helps control TRAF6 by tagging it for degradation. When SPOP is functioning properly, it prevents TRAF6 from accumulating and slows cancer progression.

However, in HCC, SPOP is often low or mutated, allowing TRAF6 to build up and intensify NF‐κB activity, making the cancer more aggressive. The scientists identified that a specific mutation in SPOP, S119N, made it even less effective at degrading TRAF6. They confirmed with laboratory and animal experiments that restoring SPOP function reduces TRAF6 levels, suppressing the cancer.

Essentially, the study demonstrates that SPOP acts as a protector against HCC by keeping TRAF6 in check. This suggests that targeting this interaction between SPOP and TRAF6 could be a new approach for treating liver cancer, and that treatments could focus on boosting SPOP activity or directly inhibiting TRAF6.

https://onlinelibrary.wiley.com/doi/10.1111/cas.70025

